# Chemical, Nutritional and Sensory Characteristics of Six Ornamental Edible Flowers Species

**DOI:** 10.3390/foods10092053

**Published:** 2021-08-31

**Authors:** Jiri Mlcek, Anna Plaskova, Tunde Jurikova, Jiri Sochor, Mojmir Baron, Sezai Ercisli

**Affiliations:** 1 Department of Food Analysis and Chemistry, Faculty of Technology, Tomas Bata University in Zlin, Vavreckova 275, 760 01 Zlin, Czech Republic; plaskova@utb.cz; 2 Institute for Teacher Training, Faculty of Central European Studies, Constantine the Philosopher University in Nitra, Dražovská 4, 949 74 Nitra, Slovakia; tjurikova@ukf.sk; 3 Department of Viticulture and Enology, Faculty of Horticulture, Mendel University in Brno, Valtická 337, 691 44 Lednice, Czech Republic; jiri.sochor@mendelu.cz (J.S.); mojmir.baron@mendelu.cz (M.B.); 4 Department of Horticulture, Agricultural Faculty, Ataturk University, Erzurum 25240, Turkey; sercisli@gmail.com

**Keywords:** edible flower, antioxidant, bioactive compound, phenolic, flavonoid, mineral element, sensory evaluation

## Abstract

Ornamental edible flowers can be used as novel nutraceutical sources with valuable biological properties. The purpose of this study was to establish nutritional, chemical, and sensory characteristics, antioxidant capacity (AC), and the relationship between their bioactive components and AC. The selected flowers *Begonia × tuberhybrida*, *Tropaeolum majus*, *Calendula officinalis*, *Rosa*, *Hemerocallis*, and *Tagetes patula*, can be easily collected due to their larger size. Their methanolic extracts were spectrophotometrically determined for polyphenols, flavonoids, and AC. Mineral elements were analyzed by atomic-absorption spectroscopy; crude protein was quantified by the Kjeldahl method. Eventually, 30 panelists evaluated sensory properties in 11 attributes. In addition, this study may serve to popularize selected blossoms. In flowers the contents of minerals were in this order: K > Ca > P > Mg > Na > Zn > Mn > Fe > Cu > Mo. AC ranged between 4.11 and 7.94 g of ascorbic acid equivalents/kg of fresh mass. The correlation coefficients between AC-total phenolics and AC-total flavonoids were *r* = 0.73* and *r* = 0.58*, respectively. It is also possible to observe a strong correlation between mineral elements and bioactive compounds. *Hemerocallis* was rated as the best and most tasteful; additionally, it exhibited the highest AC, total phenolic and flavonoid contents.

## 1. Introduction

In recent years, edible flowers (EFs) have gained more attention due to their potential as a functional food with health benefits. This increased interest is also because customers are increasingly demanding in terms of nutrition. Flowers used in the human diet must be edible, which means harmless and non-toxic; thus, blossoms treated with chemical pesticides are unfit for consumption [1,2].

The primary gastronomic use of blossoms stems from their alluring colour; the assortment of EFs includes several dozen species with a significant number of shapes, colours, and flavours [3,4]. According to Lu et al., the number of EFs varies depending on region, but roughly 97 families, 100 genera, and 180 species are found worldwide [5]. Popular edible ornamental flowers are begonia, borage, calendula, carnation, cornflower, daylily, chrysanthemum, hibiscus, nasturtium, pansy, rose, tulip, and others. In addition to the ornamental flowers described above, EFs include several vegetables (broccoli and cauliflower), herbs (common sage, chives, marjoram, mint, thyme, and summer savory), or the blossoms of some fruit trees (elderberry and apple).

Nowadays, EFs are used in cuisine for flavour, garnish, and improved nutritional value of food, but they also represent a new opportunity for gastronomic innovations [6,7]. Flowers can be consumed in various forms, including fresh, dehydrated, lyophilised, cooked, and candied. Common uses for them are salads, sauces, jellies, soups, meat dishes, dyes, beverages, ice cubes, syrups, desserts, and cakes [8,9,10].

The main component of blossoms is water; its content ranges from 70 to 95% [11]. The content of nutrients like lipids, proteins, carbohydrates, and vitamins is similar to those in vegetables [12]. However, the mineral content of EFs shows the most significant variability regarding nutrient composition, as it is affected by the micronutrients in the soil [13]. The beneficial compounds for human health include antioxidants, vitamins, phenolics, carotenoids, flavonoids, minerals, and others [14]. Moreover, bioactive substances high content represents a beneficial component of the diet because of the possible development of valuable nutraceuticals [15]. However, the recommended limits of toxic agents must be regarded to utilise flowers as food [12].

Some edible flowers are traditionally used as medicinal herbs, and several previous studies revealed their biologically active molecules with potential health effects [16]. For example, these bioactive compounds can lower the risk of cardiovascular and cancer diseases, and they additionally have many beneficial properties like anti-inflammatory, antibacterial, antidiabetic, diuretic, antifungal and others [17,18,19,20,21,22,23,24,25]. The positive efficacy in lessening the risk of some diseases is due to these molecules’ ability to reduce the damage induced by reactive oxygen species (ROS) [26]. These bioactive molecules probably may have a prolonged effect inside the body, as significant antioxidant activities (AA) have been found after the digestion in vitro of selected blossoms [27].

For example, the importance of AA in roses, begonias, and nasturtiums was mentioned by Friedman [28]. Because of the inclusion of polyphenols and ascorbic acid, nasturtium flowers demonstrated an exceptional tendency to exhibit unstable and highly reactive free radicals [29,30]. High values of AA recorded in extracts of rose flowers show a significant inhibitive effect on ROS [31,32,33]. In daylily extracts, intense scavenging ROS activity and lipid peroxidation were also observed [34,35]. *C. officinalis* extract had stronger AA when it came to scavenging free radicals than the synthetic antioxidant butylhydroxytoluene, commonly used as a food additive [18]. 

The utilisation of flowers for the human diet is associated with higher demands on their quality, sensory and nutritional properties [1] (Osimitz, Franzosa, Maciver, & Maibach, 2008). The larger size of blossoms with a simple collection is currently preferred if they can be economically effectively used [12]. The popular ornamental EFs of our gardens, specific in their size, colour, or aroma, could be convenient for the above properties and increase edible flowers’ consumption. However, the sensory properties of selected cultivars of ornamental EFs are not sufficiently described in the literature. Additionally, only a small amount of EFs have been studied, and further research is needed to use them effectively. All flower samples in this research were non-toxic, allowing them to be included in human nutrition; nevertheless, it should be noted that the daily limit of intake for some of them is yet unknown [14]. 

The study aimed to investigate the nutritional composition, total phenolics and flavonoids contents, antioxidant capacity and mineral elements (P, K, Ca, Mg, Na, Fe, Mn, Cu, Zn, and Mo) of selected EFs. This work was supplemented and extended by studying the relationship between bioactive compounds and antioxidant capacity to assess their potential benefits to human metabolism. Furthermore, the sensory properties (appearance, fragrance, consistency, acid, bitter, astringent, sweet, spicy, overall taste, juiciness, and overall evaluation) were evaluated and described.

## 2. Materials and Methods

### 2.1. Plant Material

During the period of 2018–2019, the examined plants were grown in an unheated greenhouse on the plots of experimental orchards belonging to the Mendel University in Brno. These grounds are situated in the south-eastern part of Czechia at an elevation of 170 m above the sea level in Lednice town. Besides, the average yearly temperature and precipitation are 9.2 °C and 516 mm, respectively. The soil type was classified as black soil; the value of pH/KCl is 6.8. The agrochemical attributes of the used soil are shown in Table 1 [36]. 

The criteria for selecting suitable ornamental flowers for our experiment include well-known ornamental edible species with larger blossoms that can be easily collected. The six selected flowers varied in species and colour, namely a pink cultivar of *Begonia × tuberhybrida*, a red cultivar of *Tropaeolum majus*, an orange cultivar of *Calendula officinalis*, a light yellow to a cream colour cultivar of *Rosa*, *Hemerocallis* cultivar with two-coloured petals (yellow and red), and orange cultivar of *Tagetes patula*. Some of their characteristics as shown in Table 2. 

### 2.2. Preparation of Samples

Flowers were collected in full ripeness from five randomly chosen plants of each species (cultivar). The degree of full readiness of flowers was determined from the blossom size, opening and colouring [37]. Five flowers from each cultivar were mixed and used for analyses. 

The flowers of the unique cultivar were processed promptly after harvest (within 24 h at the latest). The reaped flowers were ground in a disc type mill SJ 500 (MEZOS, Hradec Králové, Czechia). Finally, the average sample was obtained by dividing pureed samples into quarters. Each parameter was measured in five replications. The obtained data were expressed as the average of a two-year experiment (2018–2019). 

### 2.3. Extraction of Samples

The extraction of samples was performed according to the method developed by Kim et al. [38] and modified by Barros et al. [39]. The fresh flowers (10 g) were homogenised for 10 s in methanol; the plant and solvent ratio was 1:10 (*w*/*v*). The subsequent slurries were left in a water bath at a constant temperature of +25 °C for 24 h. The exact amount of methanol (100 mL) was used twice to extract residues. Both portions of methanolic extracts were combined, and the final solution was concentrated through evaporation at +40 °C to dryness (rotary evaporator R-215, Buchi Ltd., Oldham, UK). The samples were redissolved in methanol at a 100 g/mL concentration and kept at +4 °C for further utilisation. 

### 2.4. Total Phenolic Content Assay 

Total phenolic content (TPC) was measured by the method presented by Kim et al. with some modifications [38]. The sample (500 μL of extract) was brought quantitatively into a volumetric flask and diluted with distilled water to a volume of 50 mL. Then Folin-Ciocalteu’s reagent (2.5 mL) and 20% solution of sodium carbonate (7.5 mL) were added to the sample. The resulting absorbance was measured at 765 nm against a blank on UV/VIS spectrophotometer LIBRA S6 (Biochrom Ltd., Cambridge, UK). TPC in different methanolic extracts was calculated and reported as g of gallic acid equivalents (GAE) per kg of fresh mass (FM). 

### 2.5. Antioxidant Capacity by the DPPH Test Assay

Total antioxidant capacity (TAC) assay was carried out according to Brand-Williams et al. [40,41] with some modifications to analyse the antioxidant capacity of given samples [40,41]. The determination of free radical scavenging activity of methanolic flower extracts on 2,2-diphenyl-1-picrylhydrazyl (DPPH) free radical was estimated. The preparation of the stock methanol solution of DPPH and then the working solution was performed according to the procedure described by Rop et al. [14] using a spectrophotometer (LIBRA S6). In this method, 150 µL of flower extract was allowed to react with 2.85 µL of the DPPH methanolic solution. After 1 h in the dark, the absorbance was measured at 515 nm, then the values were converted using a calibration curve and expressed as g of ascorbic acid equivalents (AAE) per kg of FM [42].

### 2.6. Ferric Reducing Antioxidant Power Assay

The total antioxidant potential was estimated spectrophotometrically using the ferric reducing antioxidant power (FRAP) assay determined by Benzie and Strain with a slight modification [43]. The FRAP reagent was prepared from sodium acetate buffer (300 mM/L, pH 3.6), 10 mM/L TPTZ solution in 40 mM/L HCl and 20 mM/L FeCl_3_ solution in proportions of 10:1:1. The sample (50 µL) was added into a testing tube with 4 mL of FRAP reagent, and its absorbance was measured at 593 nm after 10 min of incubation. The standard curve was prepared using different gallic acid concentrations; the results were expressed as g GAE/kg of FM.

### 2.7. Total Flavonoid Content Assay

Total flavonoid content (TFC) was determined with the aluminium chloride colourimetric assay described by Singleton et al. [44]. Into a microcentrifuge tube, 0.3 mL of the flower extract, 3.4 mL of 30% ethanol, 0.1 mL of sodium nitrite (c = 0.5 mol/L) and 0.15 mL of aluminium chloride hexahydrate (c = 0.3 mol/L) were put and mixed. After 5 min of incubation, 1 mL of sodium hydroxide (c = 1 mol/L) was added to the mixture. The absorbance of samples was measured against the blank at 506 nm using the LIBRA S6 spectrophotometer. TFCs were calculated from the calibration curve using rutin as a standard and were expressed as g of rutin equivalents (RE) per kg of FM.

### 2.8. Dry Matter and Mineral Content Assay

The dry matter (DM) and the mineral content were measured using modified methods described by Higson and Novotny [45,46]. The plant samples were dried in a laboratory oven Venticell 111 (BMT, Brno, Czech Republic), at 105 ± 2 °C to a constant weight. The dried samples’ weights were measured and expressed as a percentage of weight concentration (*w*/*w*). 

The next step was homogenisation of dried flowers to a particle size of up to 1 mm using a SJ500 laboratory grinder (MEZOS, Hradec Kralove, Czech Republic). About 1 g of DM of the homogenised sample was mineralised with concentrated sulphuric acid and 30% hydrogen peroxide in digestion tubes using a Bloc-digest M 24 apparatus (JP Selecta, Abrera, Spain). The digested samples were quantitatively added into a volumetric flask and then diluted to a final volume of 250 mL with double-distilled water. 

The flower mineralizate was measured using an atomic absorption spectrometer PHILIPS PU 9200× with flame atomisation (Philips, Eindhoven, The Netherlands). A spectrophotometer Libra S6 was used to analyse the amount of phosphorus in the mineralizate quantitatively. The sample was prepared in a 100 mL volumetric flask, where 10 mL of the mineralizate and the same volume of ammonium-vanadomolybdate reagent were mixed. Lastly, samples were diluted up to a total volume of 100 mL with redistilled water and measured at 410 nm wavelength. For preparing the stock standard solution, potassium dihydrogen phosphate was used. The average contents of mineral elements were expressed as mg/kg of FM. 

The content of total nitrogen was established according to the Kjeldahl method (ISO 1871:2009) using the analyser unit Kjeltec™ 2300 (Foss, Hillerod, Denmark). This apparatus provides automatic distillation and approves colourimetric titration. The crude protein in g/kg of FM was estimated by multiplying the determined nitrogen content by the standard default conversion factor of 6.25 [47].

### 2.9. Sensory Analysis 

The sensory evaluation of edible flowers was performed by 30 panellists (trained students thanks to the course Sensory analysis and trained academic staff). They were acquainted with the monitored materials and instructed on the principles of analysis in advance. The course of sensory evaluation and equipment of the room for sensory analysis met precisely defined conditions according to the international standard ISO 6658. The sensor room at Tomas Bata University in Zlín was equipped with 12 separate evaluation boxes, placed next to each other and modified to limit contact with other evaluators. The room temperature was approximately 21 °C and was lit by artificial lighting. The assessment took place at 10:00 am, approximately 1 h (6 samples). It was recommended to take a break of approximately two minutes between the evaluations of the individual samples. Individual samples (each 3 flowers from one species) were administered in order *Rosa, Hemerocalis*, *Calendula officinalis*, *Begonia × tuberhybrida*, *Tagetes patula* and *Tropaeolum majus*. Before tasting, the samples were stored in reusable plastic containers at 7 °C for 12 h. Between individual samples, participants could neutralise the taste in their mouths with common water and white bread. The following sensory attributes were evaluated: appearance, fragrance, consistency, acidity, bitterness, astringency, sweetness, spiciness, overall taste, juiciness, and overall flower evaluation. The panellists assessed each blossom attribute using a 9-point hedonic scale; 1 = dislike extremely, 2, 3, 4 = subjective sense of dislike (very much/moderately/slightly), 5 = neutral, 6, 7, 8 = like slightly/moderately/very much, 9 = like extremely for overall taste and overall evaluation. They also determined the perceived intensity of each taste (acidity, bitterness, astringency, sweetness, and spiciness); 1 = very strong, maximum, 5 = slightly, moderate, 9 = without the taste. The scales for the remaining descriptors were as follows: 1 = unacceptable, 5 = neutral, 9 = ideal for appearance (suitability for food decoration); 1 = very intense and unpleasant, 5 = odourless, 9 = very intense pleasant for fragrance; 1 = very stiff, 5 = ideally crispy, 9 = flowable for consistency; 1 = dry, 5 = moderately juicy, 9 = watery for juiciness. The results were expressed graphically as the mean values of all ratings for each component and the overall score.

### 2.10. Statistical Analysis

Microsoft Office-Excel 2013 (Microsoft Corporation, Redmond, WA, USA) and STATISTICA CZ version 12 (StatSoft, Inc., Tulsa, OK, USA) were used for data analysis. The results were expressed by mean ± standard deviation (M ± SD). To establish statistically significant differences between individual species, Shapiro-Wilk test of normality and Levene’s test of homogeneity of variances was performed. Since the conditions for the calculation by ANOVA analysis were not complied in any of the monitored data sets, a non-parametric Kruskal-Walllis test (α = 0.05) were performed. Correlation functions were calculated using statistic software Unistat 5.1 (Unistat Ltd., London, UK) and Microsoft Office-Excel 2010 (Microsoft Corporation, Redmond, WA, USA).

## 3. Results and Discussion

The results of the chemical analyses are shown in Table 3, Table 4, Table 5, Table 6, Table 7 and Table 8. The results were expressed as an average of a two-year experiment (2018–2019) since there was no statistically significant difference between the years in any parameter researched.

### 3.1. Total Content of Phenolic Substances, Total Antioxidant Capacity and Total Flavonoid Content

Table 3 provides TPC values of six edible flowers. These TPC values varied from 3.23 g GAE/kg in *T. majus* to 6.59 g GAE/kg in *Hemerocallis*, with almost double the difference. The results of *Hemerocallis* showed up to six times higher TPC than in some cultivars of daylilies presented by Muchová [48]. 

The TPC values for tested pink *Begonia × tuberhybrida* were 4.82 g GAE/kg of FM. They were similar for the white cultivar of Begonia (*B. semperflorens* Link et Otto) [49] and double higher when compared to another pink cultivar, ‘Chanson‘ [48]. 

TPC in *T. patula* (French marigolds) was 4.78 g GAE/kg of FM, and this content is slightly higher than that reported by Rop et al. [14] but lower than in different orange cultivars of French marigold flowers [50]. 

According to Ashraf et al., for *C. officinalis*, the TPC values were slightly lower (by 0.83 g) than the values measured by us (3.65 g GAE/kg FM), which could be caused by the fact that other parts of the plants (leaves) were used or different growing conditions [51]. Ferreira et al. quantified TPC values of 2.30 g GAE/kg DW in calendula flowers [52]. This research found an aqueous methanol solution (70:30, Me-OH:H_2_O) more effective for extracting phenolic compounds than pure methanol; the values were probably higher because the solvent was highly polar and had the highest dielectric constant [52].

The content of total phenolic substances in *Rosa* was determined to be 4.45 g GAE/kg FM, which is approximately 1.12 times lower than that measured by Rop et al. [14]. Yang and Shin found the difference between TPC in ethanolic samples of roses, and their values ranged from 7.99 to 29.79 g/kg FM [1]. They also reported that yellow and pink roses had lower TPC than red roses. Despite different flower varieties and conditions of extraction, these values are lower than those reported by Gonçalves et al., where the orange rose cultivar had a slightly higher TPC content (17.60 g GAE/kg FM) than *Tagetes erecta L.* (17.47 g GAE/kg FM) [11]. The considerable variation could indicate that the composition of substances differs within the genus.

As mentioned above, the red cultivar of *T. majus* exhibited the lowest TPC value (3.23), which is significantly lower than the value for the orange cultivar exhibited by other authors [29,53]. Nevertheless, our TPC results are comparable to those reported by Rop et al. [14] and higher than those reported by Muchová [48]. The differences could be caused by using a different variety, growing conditions, the flower’s measured parts, or its adjustment before analysis.

The total phenolic range level is comparable with some berries *Vaccinium L. hybrids* and *Rubus L. species* [54,55], both of which are considered to be great sources of antioxidants [29]. This content is higher when compared to different vegetables like Ceylon spinach, white and red onions [56], lamb’s lettuce [57], or other green leafy vegetables [58]. Flowers may be ideal for making salads more appealing to consumers, either adding colour or increasing the phenolic content of the food. 

In this study, total flavonoids were another parameter studied in flowers, as seen in Table 3. Flavonoids are likely the most significant natural phenolics, and also they are one of the most varied and widespread natural chemicals [59]. The TFC in the flowers ranged from 1.29 to 3.76 g RE/kg FM. The highest TFC was determined in *Hemerocallis* (daylily), and the lowest was observed in the red variety of *T. majus*. For other flowers, the TFC was above 2 g of RE/kg of FM. In the Rosa’ Gloria Dei’, the total flavonoid contents were 2.43 g of RE/kg of FM, which is slightly higher than that observed in Rosa odorata [14]. According to a study by Yang and Shin, the TFC of edible roses ranged between 0.79 to 5.32 g/kg FM; our analysed cultivar is in this range [1]. The flowers reached significantly higher TFC values than some vegetables and fruit, such as tomatoes (0.133 to 0.474 g RE/kg FM) [60], watermelons (0.09 to 0.27 g RE/kg FM) [61], or fruit studied by Mirzaei et al., such as blackberry and black grape, whose TFC values ranged from 0.05 to 1.03 g RE/kg FM [62]. According to studies on 12 cultivars of EFs, the yellow blossoms had a higher content of flavonoids and suggested that they have a stronger antioxidant potential than other colours [59]. This correlates with our results because the flowers with the highest TFC content were *Hemerocallis* and *Rosa* with yellow colour petals. However, Garzón et al. analysed the *T. majus* flowers depending on their colour. The yellow cultivar had lower TPC values and antioxidant activity than the orange and red cultivars due to the low content of primary anthocyanins [29]. 

Further research could involve using high-performance liquid chromatography (HPLC) to identify and accurately quantify phenolic compounds in the sample. In the case of including HPLC analysis in this study, its length and complexity would exceed the proposed research framework.

The antioxidant potentials of flower extracts were estimated using two different colourimetric assays in vitro based on electron-transfer reactions. The first DPPH method was measured antioxidants’ capacity to scavenge an organic radical; the results were expressed as ascorbic acid equivalents. The second FRAP method was estimated antioxidants’ ability to reduce ferric to the ferrous ion, and findings were reported as reducing power per gallic acid equivalent. Combining these two approaches based on distinct mechanisms may provide more reliable and complex data for antioxidant capacity. A single spectrophotometric assay may not provide satisfactory results because of its deficiency of specificity and sensitivity [63]. Both methods are widely used because of their simplicity, speed, high reproducibility, and ability to measuring by simple instrumentation [41,64,65]. Each of them has some advantages and limitations. For example, the DPPH assay can detect weak antioxidants and thermally unstable compounds; however, DPPH might react with other radicals in the sample and is sensitive to light [43,64,65]. The FRAP method result may not positively correlate with the total antioxidant activity of the sample; because this assay is non-specific and has limitations in measuring slow-reacting polyphenolic antioxidants and thiols [43,66].

The total antioxidant capacity of samples ranged from 4.11 g AAE/kg FM in *C. officinalis* to 7.94 g AAE/kg FM in daylilies. TCA values above 6 g AAE/kg FM were measured in *T. patula*, *Rosa*, and *T. majus*. The *Hemerocallis* (daylily) achieved a higher antioxidant capacity than the edible flowers in the Rop et al. study; the TAC of edible flowers ranged from 4.21 to 6.96 g AAE/kg FM [14]. In addition, the strong antioxidant activities of daylilies extracts (aqueous and ethanolic) were observed by Que et al. [35]. These results exhibited lower AA than synthetic antioxidant (butylated hydroxyanisole) but higher than α-tocopherol. According to Fu et al., the highest antioxidant capacity and the highest proportion of phenolic substances is in the opening stage of daylilies [17]. Mao et al. found that the use of lyophilised daylily flowers is more suitable for obtaining an extract with higher AA and a higher TPC than the use of flowers dried with hot air [67]. The limitation of using daylily is that each flower only lasts one day. The flower extracts from *T. majus* are active, reducing agents, which indicates a good ability to scavenge radicals [68]. According to Pavithra et al., the methanol extracts of flowers have scavenging abilities dependent on their concentration (25 mg/mL and higher) but lesser than ascorbic acid [69].

Comparing the results obtained from TAC-FRAP with TAC-DPPH, it is evident that the extracts’ ability to reduce Fe^3+^ has a different order than the ability to quench the DPPH^•^ radical. Additionally, the values obtained by FRAP assay show that the highest antioxidant capacity corresponded to *T. patula* (5.62 g GAE/kg FM), followed by *Begonia × tuberhybrida* (5.15 g GAE/kg FM) and *T. majus* (4.98 g GAE/kg FM). The lowest AC was that of *C. officinalis* (3.44 g GAE/kg FM). The FRAP values displayed a 1.6-fold difference. 

People generally do not consume as many edible flowers as carrots, radishes, cucumbers, tomatoes, and other vegetables. Because some edible flowers have a pungent or strong aroma, it is advisable to use them sparingly to encourage food flavour [70]. The ornamental EFs evaluated in the study were non-toxic; nevertheless, it should also be considered that the daily limit for their ingestion is not determined for all samples, and no international authority has published the official list of EFs [12,71,72]. Consumption and culinary use of some EFs were documented in history before May 1997; consequently, these flowers are not defined as novel foods [73,74]. For example, none of the species analysed in our research was featured on official lists like the Novel Food Catalogue [72]. On the other hand, other blossoms that cannot prove their significantly large consumption by people before 15 May 1997 must be submitted to the European Food Safety Authority for their safety application as novel foods [73,75].

Lucarini et al. [75] examined available information in databases and bibliographies about the same plant genera as our study, and they discovered no scientific proof that these plants constitute potential allergens. 

Even the most favourable herbs can have unpredictable effects, for example, the consumption of more than 39.5 g of fresh *T. majus* flowers surpassing the daily erucic acid allowance [70,76]. The number of blossoms consumed may be the limiting factor because of allergic and toxic reactions by sensitive persons to some of the flower unidentified compounds [14]. In addition, pollen from specific blossoms might induce an allergic response in humans with allergies or asthma [75]. Thus, it is important to study the toxicity of EFs with high antioxidant activity to establish their safety as food additives. Moreover, identification of the plant is critical because some toxic flowers could be readily confused with EFs, such as daylilies with true lilies, and confusing them might be dangerous [77].

### 3.2. The Content of Mineral Elements

Five macroelements (P, K, Na, Ca, Mg) and five microelements (Fe, Mn, Cu, Zn, Mo) were determined and quantified in the petals of diverse species of ornamental edible plants. These mineral elements are essential for the human body’s vital functioning, but the available literature contains scant data about their content in EFs. The contents of minerals, expressed on a FM basis, are shown in Table 4 and Table 5, and were in this order: K > Ca > P > Mg > Na > Zn > Mn > Fe > Cu > Mo. The macroelements amount ranged from 121 to 3623 mg/kg FM (Table 4), and the content of microelements then from 0.98 to 14.91 mg/kg FM (Table 5). 

*Hemerocallis* had the greatest Ca, Mg, Fe, Mn, Cu, and Mo concentrations, whereas *T. patula* contained the highest amount of K, Na and Zn, and the highest P was detected in *T. majus*. In contrast, the lowest P, K, Mg, Na and Fe content was observed in *Begonia × tuberhybrida*; *Rosa* had the least amount of Ca, Mn and Zn, the lowest quantity of Cu and Mo was found in *T. majus*. 

The content of mineral elements is comparable to the flower mineral concentration mentioned by Rop et al. [14]. When compared to ordinary fruit and vegetables, EFs are a good source of minerals. This is evidenced by the higher K content than vegetables and fruit, which have an average K content of 1500–2100 mg/kg FM (Table 4) [78,79,80]. Several researchers observed a similar trend in which potassium content was highest in flowers [14,81,82,83]. Potassium content in flowers was higher than in leaves, roots, and stem of *Chrysanthemum indicum* L. [82]. According to Grzeszczuk et al., in other Hemerocallis species, the most abundant macroelement was K, which correlates with our results, but P content was higher than that of Mg [83]. However, Navarro-González et al. reported that *T. majus* and *Tagetes erecta* blossoms contain more zinc, iron, and manganese than potassium [53]. Flowers (100 g fresh weight) provided only 10.0–18.1% of the daily recommended K intake of 2000 mg for adults [47]. Potassium content is an important source for maintaining acid-base balance in blood and tissues and preventing cardiovascular or oncogenic diseases [84].

The content of other elements in flowers is comparable to vegetables [80], but some selected leafy vegetables had a higher content of sodium than potassium [58]. Compared to fruit, a two-fold increase in Ca and Mg contents and a fourfold rise in Na content can be observed [78,85,86]. In addition, the content of mineral elements in flowers can be compared with published minerals data about wild-growing and cultivated mushrooms. Calcium and sodium contents are two to four times higher than that of fungi, the content of other elements is approximately comparable, but the phosphorus one is twice lower [87,88]. 

Mineral elements perform several functions: as components of enzymes, regulation of cellular energy transduction, gas transport, antioxidant defense, membrane receptor functions, second-messenger systems, and integration of several physiological functions [89,90,91]. Furthermore, they can strengthen the immune system [92,93], form building blocks of the human skeleton [91,94] and are associated with anti-inflammatory [24,95], antibacterial [93,96], antifungal [97] and antiviral effects [98]. A few published research papers deal with the content of mineral elements in EFs regarding their relevance for human health [14,83,99,100].

Previous research has shown that iron concentrations in ornamental flowers are highly varied, compared to our results, for example, *Begonia boliviensis* with lower content of 2.65 mg/kg FM [14], *T. majus* with slightly lower content from 5.51 to 6.47 mg/kg FM [14,53], and *T. erecta* with slightly higher amount of 10.26 mg/kg FM [53]. Different species probably caused variations in mineral elements content between the flower samples because they were grown in the same location and with identical agricultural practices.

All analysed flower species have high molybdenum levels based on recommended daily intakes for adults (50 µg) since 100 g of fresh blossoms provides 64–196% of this value [47]. The concentration of Mo affects ascorbic acid level; for example, its deficiency can cause a decrease in AA content in some vegetables [101]. Tolerable upper intake level of Mo range from 0.1 to 0.6 mg/day [102]; therefore, consuming a slight amount of flowers is unlikely to be a risk for human health. *Hemerocallis* can be considered as a possible source of Cu (0.29 mg/100 g FM), Mn (0.88 mg/100 g FM), and Zn (1.15 mg/100 g FM), and these mineral elements can contribute to daily recommended dietary allowances for adults. For example, 100 g fresh *Hemerocallis* can provide 29.3% copper, 43.8% manganese and 11.5% zinc for dietary reference intakes [47]. EFs should not be overlooked as a source of mineral elements in the human diet; however, it is unlikely that somebody would eat 100 g of flowers in a single day. Edible flowers will most likely be used as a garnish to add colour and flavour to the food.

### 3.3. Dry Matter and Content of Crude Protein

The dry matter and the content of crude protein of the edible flowers are shown in Table 6. As can be seen from the results, the DM of these edible flowers ranged from 7.38 to 14.39%, and this amount is slightly higher than the average content in fruit and vegetables [103]. On the other hand, according to Montañés Millán et al. [104], the DM percentage in blossoms from the fruit tree was higher. When comparing our DM results to previous research for the same plant genus, *Begonia nelumbiifolia* ranged from 5.31 to 6.15%, which is lower than Begonias results in our experiment [105]. However, Rop et al. determined *Begonia boliviensis* (14.20%) with a higher DM [14]. In addition to the last-mentioned research, they determined a higher DM for *T. patula* (9.68%) and *T. majus* (11.27%) and lower DM for *Rosa odorata* lower DM (10.09%) [14]. De Lima Franzen et al. observed a higher DM percentage for rose (*Rosa × grandiflora*) and *C. officinalis* of 15.44% and 10.66%, respectively [106]. 

The CP content of EFs samples was estimated by the Kjeldahl method, and the results ranged between 2.89 to 4.56 g/kg of FM (Table 6). 

The highest values were reached for *T. majus* (4.56) and *Begonia × tuberhybrida* (4.51). Comparing these CP values with results obtained by Rop et al. [14], *T. majus* had slightly lower values (4.74 g/kg FM), and Begonia had one and a half times higher than another cultivar. However, the CP contents of *T. majus* and other varieties of Begonia and roses cultivated in Japan were significantly higher than in our research [9]. The difference may be caused by different cultivars, place and growth conditions. A similar CP was observed in EF *Fernaldia pandurate* with 3.0 g/kg FM [107]. This protein content can be comparable to some fruit and vegetables but not to cereals because they have an order of magnitude higher content [79]. Similar proportion content was observed in some fruit, for example, plum with 3.9 g/kg FM [108] or red banana (*Musa acuminata*) [109]. The CP content in fresh vegetables was higher than in our experiment, for example, radishes with 5 to 15.5 g/kg, beetroot with 13.22–14.43 g/kg [110], celery with 6.91 g/kg, carrot with 5.64 g/kg, and turnip with 4.88 g/kg [108]. Thus, flower petals could not be regarded as good protein sources because of their low CP levels [106]; also, people consume fewer EFs than radishes, carrots and other popular types of vegetables.

### 3.4. Correlation Analysis between Mineral Elements and Bioactive Compounds

The correlation coefficients of mineral elements and bioactive compounds in edible flowers are shown in Table 7 and Table 8. Significantly strong positive correlations were observed between some mineral elements contents; for example, the correlation of Na-K (*r* = 0.92 **), Zn-K (*r* = 0.96 **), Zn-Na (*r* = 0.92 **) and Zn-Fe (*r* = 0.83 **). Furthermore, considerable high positive correlations between TFC-Mo (*r* = 0.93 **) and TFC-Cu (*r* = 0.81 *) were found. From a different point of view, negative relationships were noticed between the contents of M and P (*r* = −0.88 **), between TFC and P (*r* = −0.69 *), and also between Cu and P (*r* = −0.59 *).

Table 8 shows the selected correlation coefficients between TAC-DPPH and mineral elements, TPC or TFC. These relationships are studied to assess if these components contribute to the TAC of the flowers and if they have any potential benefits for human metabolism.

In accordance with some research studies [111,112,113,114], significant correlations between TAC, TPC and TFC were commonly achieved in our results as well, from *r* = 0.57 * to 0.94 **. The results imply that blossoms with a higher amount of polyphenols have a stronger antioxidant activity, and flavonoids comprise an important group of phenolic compounds. Some authors also found a strong positive correlation between TPC and FRAP assay values [43,71,115]. The antioxidant activity could be attributed to some mineral elements like copper, iron and manganese [116]. In our case, AC correlates with the Ca (*r* = 0.68 *) and Fe (*r* = 0.61 *), which means a moderate positive correlation; some authors also described these relationships [117,118]. Their articles state the importance of nutrition by given elements on the content of bioactive substances. On the other hand, the correlations between TAC and the remaining mineral elements were weak or negligible. Aside from polyphenols, the antioxidant activity of floral extracts may be affected by other biological compounds, including vitamins, pigments such as carotenoids, mineral elements, nitrogenous compounds, and other metabolites. [17,119,120,121].

### 3.5. Sensory Evaluation

Necessary criteria for evaluating the quality of edible flowers for gastronomy are organoleptic performance, flavour, and overall impression. The overall evaluation of the flowers makes a positive impression on consumers. In our research, the best-rated flowers were *Hemerocallis* with 8.2 points, followed by *T. majus* (7.4), *Begonia × tuberhybrida* (7.3), *C. officinalis* (7.2), *Rosa* (6.7), and *T. patula* (6.2). 

**Figure 1:**
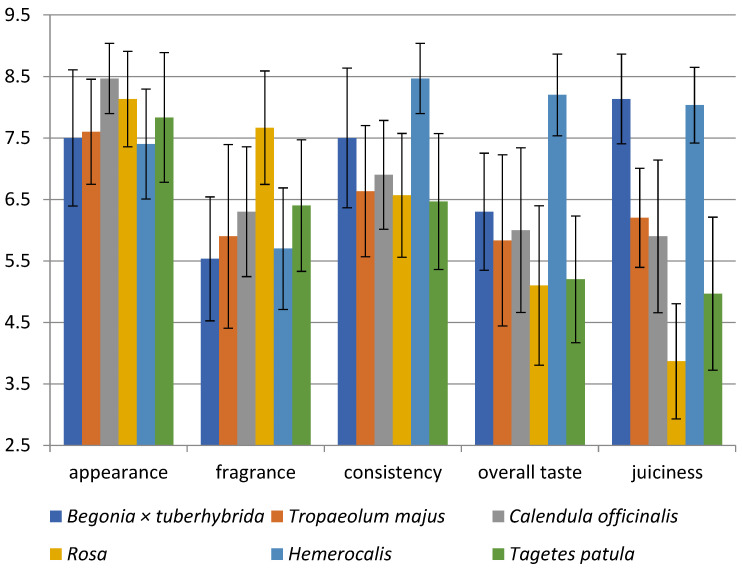
Sensory analysis diagram for six edible flowers; *Begonia × tuberhybrida*; *T. majus*; *C. officinalis*; *Rosa*; *Hemerocallis*; *T. patula*.

As can be seen in Figure 1, the appearance of all analysed flowers was evaluated as suitable for decorating dishes; blossoms of *C. officinalis* (8.5) appeared to be the most acceptable. The difference between the species was 1.1 points. According to Kelley et al., the colour and composition of flowers are important characteristics influencing consumer preferences [3]. For example, the nasturtium mixture containing flowers of darker colours such as orange and crimson was evaluated as more appealing [3,122]. In addition, the colour of EFs can influence sales because of the appeal to the appetite of consumers; for example, red flowers may increase appetite, the yellow one can elicit happiness, and the orange expresses affordability [122]. Furthermore, the customers can associate the colour of EFs with the taste of food in the same hue [122].

The fragrance of blooms was evaluated in all samples as pleasant with various levels of intensity. *Rosa* ‘Gloria Dei’ was identified as the flower with the most pleasant scent (7.7) because the scale ranged from very intense pleasant (=9) through odourless (=5) to very unpleasant fragrance (=1). The other blossoms were rated from 5.5 to 6.4 points, corresponding to a lower intensity scent. The fragrance may attract consumers by arousing their interest in the flowers, but the buds are generally odourless; thus, only fully ripeness blossoms were collected [123]. Therefore, the petals are the main source of aromatic compounds in many flowers; for example, the petals of *Rosa damascena* are used as a source of aroma and natural scents [107]. 

In evaluating consistency, the crispness is related to the water content because the cells or cavities are exhibited this property when they are filled with air instead of water [124]. The remaining *Hemerocallis* (8.5) and *Begonia × tuberhybrida* (7.5) were evaluated as more flowable. Another parameter related to consistency is probably juiciness due to evaluating these two EFs as watery and more flowable. The level of juiciness significantly varied. For example, *Rosa* (3.9) was evaluated as drier, and the remaining flowers showed a subjective degree of juiciness or watery. The sensory properties of the rose, such as its dryness and crispiness, can be influenced by the high DM content (14.39% *w*/*w*).

The evaluation of the overall taste of flowers is important for their acceptance and valuation as food. *Hemerocallis* flowers have an 8.4-point gain, which means delightful taste. On the contrary, *Rosa* (5.1) and *T. patula* (5.2) flowers were evaluated neutrally; for instance, they can be more suitable as garnish. The remaining flowers had a slightly pleasant overall taste.

Statistically significant differences were found in: appearance (*C. officinalis* vs. *Begonia × tuberhybrida* and *T. majus* and *Hemerocallis*), fragrance (*Rosa* vs. all species), consistency (*Hemerocallis* vs. all species, *Begonia × tuberhybrida* vs. *T. patula and Rosa)*, overall taste (*Hemerocallis* vs. all species, *Begonia × tuberhybrida* vs. *Rosa* and *T. patula*), juiciness (*T. majus* vs. *C. officinalis*, *Hemerocallis* vs. *Begonia × tuberhybrida*, *T. patula* vs. *Rosa* and *C. officinalis* and *T. majus*) (*p* < 0.05).

**Figure 2:**
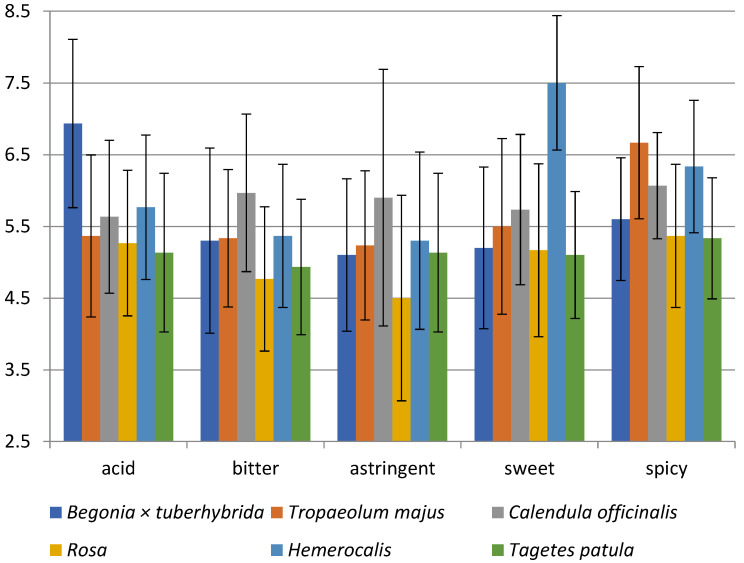
Sensory analysis diagram for the taste of 6 edible flowers; *Begonia × tuberhybrida*; *T. majus*; *C. officinalis*; *Rosa*; *Hemerocallis*; *T. patula*. The scale of taste intensity ranged from very strong, maximum (=1) through slightly moderate (=5) to without taste (=9).

Figure 2 shows the sensory analysis results on the five various taste qualities-sweet, acid, bitter, astringent, and spicy. If the blossom received 9 points in the sensory analysis of taste, it did not contain the evaluated taste; for example, it was not sweet at all. The sourness intensity ratings were slightly acidic in all flowers; the range of gained points was from 5.1 to 6.9. Besides, *Begonia × tuberhybrida* was evaluated as the least acidic (6.9). 

Further, all blossoms were rated as slightly bitter, with bitterness perceived most intensely in rose (4.8), and *C. officinalis* with 6 points was rated as less bitter. According to Mlcek and Rop, the taste of *C. officinalis* should be slightly bitter, which corresponds with our results [12]. However, the difference is in the taste of *T. patula* because it should be bitterish or with bitter undertones [12,125].

Panellists described a similar intensity of slightly astringent taste for the evaluated flowers; for example, *Rosa* (4.5) was more astringent than *C. officinalis* (5.9). 

The next evaluated taste quality was sweetness; all flowers except *Hemerocallis* were rated similarly to slightly sweet with a point range from 5.1 to 5.7. In contrast to other flowers, *Hemerocallis* was described as unsweetened at all (7.5). Mlcek and Rop described the tastes of rose as sweet and daylily as slightly sweet, which does not correspond to the evaluators’ opinions [12].

Finally, the flowers’ spiciness was evaluated as slightly spicy, with different intensity. The blossoms of *T. majus* (6.7) tasted the least spicy; in comparison, spicier flowers were *T. patula* (5.3) and *Rosa* (5.4).

According to sensory evaluation, *Hemerocallis* had the highest score in the sensory analysis and the most acceptable overall taste. The flower buds seem to be the most widely consumed part of the daylily [12]. The *Hemerocallis* have a mild taste, albeit with a sour, astringent, and spicy touch. These flowers were not evaluated particularly as sweet, and therefore could be used as an alternative to mustard due to their peppery, radishes, and spicy taste [12,126]. However, *Hemerocallis* ‘Bonanza’ was statistically assessed as one of two non-preferred cultivars (15 daylilies) according to the taste preferences [127]. According to Grosvenor, the red (dark) varieties have more bitterness, and the tested cultivar has a yellow flower with a red centre [128]. 

Statistically significant differences were found in: acid (*Begonia × tuberhybrida* vs. all species), sweet (*Hemerocallis* vs. all species), bitter (*C. officinalis* vs. *Rosa* and *T. patula)*, astringent (*C. officinalis* vs. *Rosa*), spicy (*T. majus* vs. *Begonia × tuberhybrida* and Rosa and *T. patula*) (*p* < 0.05).

## 4. Conclusions

This study evaluated selected Czech flowers’ suitability as nutritional food with the health benefits in terms of the content of bioactive substances, mineral elements, and sensory analysis. The individual flowers are not usually consumed in large quantities but rather as a garnish or ingredient for dishes. In terms of the current popularisation of nutraceutical, edible flowers can represent a significant natural source of bioactive substances, containing a higher concentration of these than ordinary fruit or vegetables. In this research, we determined the total phenolic, flavonoids and antioxidant capacity of six ornamental flowers. Investigation of correlations confirms the findings of earlier research. The correlation study suggests that polyphenolic and flavonoids form an important part of the antioxidant compounds of these flowers. This study confirms that the amount of bioactive substances in edible flowers is affected by various factors, including the plant’s external and internal environment during growth, the time of collection, post-harvest technologies; however, optimized cultivating and harvesting protocols have the potential to standardize the produce. In the future, edible flowers can serve as a natural source for food supplements that contain these substances. Besides, the flowers presented in this study will certainly be the food industry’s future with their taste characteristics, size, and ease of collection. Many pieces of research on this topic are likely to be carried out.

## Figures and Tables

**Table 1:** Agrochemical characteristics of the soil.

Mineral Element	Content in Soil ^1^	Mineral Element	Content in Soil ^1^
Phosphorus	84	Iron	4980
Potassium	269	Manganese	560
Calcium	4989	Copper	18
Magnesium	293	Zinc	22
Sodium	55	Molybdenum	4.3

Note: ^1^ All values of mineral content are expressed in mg/kg.

**Table 2:** Species and cultivars of edible flowers used in the present experiment.

Latin Name	English Name	Cultivar
*Begonia × tuberhybrida*	Tuberous Begonia	Doublet Rose
*Tropaeolum majus*	Nasturtium	Empress of India
*Calendula officinalis*	Pot Marigold	Orange King
*Rosa*	Rose	Gloria Dei
*Hemerocallis*	Daylilies	Bonanza
*Tagetes patula*	French marigolds	Antiqua Orange

**Table 3:** Total phenolic content (g of GAE/kg of FM), total antioxidant capacity (g of AAE/kg of FM–DPPH or as g GAE/kg of FM-FRAP) and total flavonoid content (g of RE/kg of FM) in 6 species of edible flowers.

Species	TPC	TFC	TAC-DPPH	TAC-FRAP
*Begonia × tuberhybrida*	4.82 ± 0.27 ^a^	2.32 ± 0.21 ^a^	5.85 ± 0.31 ^a^	5.15 ± 0.57 ^a^
*Tropaeolum majus*	3.23 ± 0.35 ^b^	1.29 ± 0.32 ^b^	6.23 ± 0.48 ^b^	4.98 ± 0.56 ^a^
*Calendula officinalis*	3.65 ± 0.19 ^b^	2.05 ± 0.20 ^a^	4.11 ± 0.30 ^c^	3.44 ± 0.29 ^b^
*Rosa*	4.45 ± 0.20 ^a^	2.43 ± 0.18 ^a^	6.61 ± 0.41 ^b^	4.57 ± 0.31 ^a^
*Hemerocallis*	6.59 ± 0.23 ^c^	3.76 ± 0.23 ^c^	7.94 ± 0.26 ^d^	4.71 ± 0.25 ^a^
*Tagetes patula*	4.78 ± 0.44 ^a^	2.02 ± 0.17 ^a^	6.64 ± 0.38 ^b^	5.62 ± 0.28 ^c^

Note: All values are expressed as the mean ± standard deviation (SD) (*n* = 10). Values in a column that do not share the same superscript letters (^a,b,c,d^) are significantly different at *p* < 0.05. TPC: total phenolic content; TFC: total flavonoid content; TAC: total antioxidant capacity; GAE: gallic acid equivalents; FM: fresh mass; RE: rutin equivalents.

**Table 4:** Content of macroelements in 6 species of edible flowers.

Species	Phosphorus	Potassium	Calcium	Magnesium	Sodium
*Begonia × tuberhybrida*	208.59 ± 11.71 ^a^	2008.55 ± 91.02 ^a^	385.75 ± 12.33 ^a^	131.18 ± 7.66 ^a^	70.34 ± 5.28 ^a^
*Tropaeolum majus*	452.02 ± 8.23 ^b^	2353.30 ± 105.71 ^b^	317.54 ± 14.47 ^b^	152.89 ± 8.23 ^b^	85.21 ± 4.55 ^b^
*Calendula officinalis*	268.98 ± 7.89 ^a^	2988.64 ± 90.73 ^c^	294.08 ± 10.18 ^b^	189.56 ± 9.20 ^c^	86.31 ± 2.10 ^b^
*Rosa*	245.15 ± 12.32 ^a^	2033.44 ± 89.36 ^a^	285.58 ± 15.15 ^b^	142.45 ± 6.65 ^ab^	79.23 ± 4.59 ^ab^
*Hemerocallis*	235.05 ± 10.18 ^a^	2759.22 ± 90.65 ^d^	490.82 ± 9.68 ^c^	284.15 ± 4.29 ^d^	96.08 ± 3.50 ^c^
*Tagetes patula*	397.08 ± 8.94 ^c^	3623.78 ± 100.05 ^e^	362.95 ± 7.26 ^a^	203.14 ± 4.84 ^c^	121.00 ± 1.58 ^d^

Note: All values are expressed as the mean ± SD (*n* = 10). Values in the column that do not share the same superscript letters (^a,b,c,d,e^) indicate significant differences at *p* < 0.05. The content of macroelements is expressed as mg/kg of FM.

**Table 5:** Content of microelements in 6 species of edible flowers.

Species	Iron	Manganese	Copper	Zinc	Molybdenum
*Begonia × tuberhybrida*	3.12 ± 0.34 ^a^	5.77 ± 0.19 ^a^	1.28 ± 0.10 ^a^	4.78 ± 0.93 ^a^	0.76 ± 0.08 ^a^
*Tropaeolum majus*	6.52 ± 0.58 ^b^	5.79 ± 0.22 ^a^	1.22 ± 0.04 ^a^	8.89 ± 1.02 ^b^	0.32 ± 0.01 ^b^
*Calendula officinalis*	4.62 ± 0.21 ^c^	7.33 ± 0.21 ^b^	2.14 ± 0.08 ^b^	10.79 ± 0.75 ^c^	0.59 ± 0.08 ^c^
*Rosa*	4.02 ± 0.10 ^c^	3.41 ± 0.24 ^c^	2.31 ± 0.02 ^b^	4.62 ± 0.42 ^a^	0.70 ± 0.04 ^a^
*Hemerocallis*	8.70 ± 0.26 ^d^	8.75 ± 0.17 ^d^	2.93 ± 0.10 ^c^	11.45 ± 0.55 ^cd^	0.98 ± 0.09 ^d^
*Tagetes patula*	8.20 ± 0.23 ^d^	7.64 ± 0.20 ^b^	1.24 ± 0.01 ^a^	14.91 ± 1.21 ^e^	0.43 ± 0.01 ^e^

Note: All values are expressed as the mean ± SD (*n* = 10). Values in the column that do not share the same superscript letters (^a,b,c,d,e^) are significantly different at *p* < 0.05. The content of microelements is expressed as mg/kg of FM.

**Table 6:** Dry matter and the content of crude protein in 6 species of edible flowers.

Species	Dry Matter	Crude Protein
*Begonia × tuberhybrida*	11.34 ± 0.09 ^a^	4.51 ± 0.32 ^a^
*Tropaeolum majus*	7.38 ± 0.12 ^b^	4.56 ± 0.35 ^a^
*Calendula officinalis*	8.98 ± 0.09 ^c^	3.48 ± 0.22 ^b^
*Rosa*	14.39 ± 0.15 ^d^	2.89 ± 0.19 ^c^
*Hemerocallis*	11.24 ± 0.12 ^a^	3.54 ± 0.25 ^b^
*Tagetes patula*	9.34 ± 0.15 ^c^	3.01 ± 0.31 ^b^

Note: All values are expressed as the mean ± SD (*n* = 10). Values in a column that do not share the same superscript letters (^a,b,c,d^) are significantly different at *p* < 0.05. The content of dry matter is expressed as% *w*/*w*, and the content of crude protein is expressed as g/kg of FM.

**Table 7:** Correlation analysis between TPC, TAC, TFC and phosphorus (P), potassium (K), calcium (Ca), magnesium (Mg), natrium (Na), iron (Fe), manganese (Mn), copper (Cu), zinc (Zn) and molybdenum (Mo) of six edible flowers grown for two years.

	P	K	Ca	Mg	Na	Fe	Mn	Cu	Zn	Mo	TAC	TPC	TFC
P	1												
K	0.40	1											
Ca	−0.29	0.15	1										
Mg	−0.09	0.59 *	0.73*	1									
Na	0.51	0.92 **	0.24	0.57 *	1								
Fe	0.47	0.68 *	0.57	0.81 *	0.82 *	1							
Mn	0.07	0.72 *	0.67*	0.83 *	0.59 *	0.71 *	1						
Cu	−0.59 *	−0.05	0.36	0.64 *	−0.1	0.19	0.21	1					
Zn	0.49	0.96 **	0.29	0.69 *	0.92 **	0.83 *	0.81 *	0.00	1				
Mo	−0.88 **	−0.28	0.65 *	0.46	−0.3	−0.04	0.20	0.76 *	−0.26	1			
TAC	0.01	−0.03	0.68 *	0.48	0.31	0.61 *	0.14	0.31	0.11	0.39	1		
TPC	−0.52	0.12	0.88 *	0.71 *	0.24	0.45	0.47	0.58	0.18	0.82 *	0.73 *	1	
TFC	−0.69 *	0.00	0.77 *	0.72 *	0.06	0.30	0.40	0.81 *	0.06	0.93 **	0.57 *	0.94 **	1

Note: Mean values were used in the analyses of chemical parameters at levels of statistical significance (* *p* < 0.05; ** *p* < 0.01).

**Table 8:** Selected correlation coefficients between TAC-DPPH and mineral content, TPC and TFC.

Variables	Coefficient	Variables	Coefficient
TAC and phosphorus	*r* = 0.0141	TAC and potassium	*r* = 0.0316
TAC and calcium	*r* = 0.6790	TAC and magnesium	*r* = 0.4848
TAC and sodium	*r* = 0.3119	TAC and iron	*r* = 0.6093
TAC and manganese	*r* = 0.1407	TAC and copper	*r* = 0.3061
TAC and zinc	*r* = 0.1091	TAC and molybdenum	*r* = 0.3882
TAC and TPC	*r* = 0.7288	TAC and TFC	*r* = 0.5786

## Data Availability

New research data were presented in this contribution.
